# Associations of Stroke With Risk of Epilepsy: Results From the Atherosclerosis Risk in Communities (ARIC) Study

**DOI:** 10.1002/acn3.70144

**Published:** 2025-07-15

**Authors:** Jiping Zhou, Asma A. Ladak, Connor A. Law, Michelle C. Johansen, Anny Reyes, Silvia Koton, Sean Kelly, Jeubin Huang, Kamakshi Lakshminarayan, Rebecca F. Gottesman, Emily Johnson, Andrea L. C. Schneider

**Affiliations:** ^1^ Department of Neurology University of Pennsylvania Perelman School of Medicine Philadelphia Pennsylvania USA; ^2^ Department of Neurology Johns Hopkins University School of Medicine Baltimore Maryland USA; ^3^ Neurological Institute and Epilepsy Center Cleveland Clinic Cleveland Ohio USA; ^4^ Department of Nursing Tel Aviv University Tel Aviv‐Yafo Israel; ^5^ Department of Epidemiology Johns Hopkins Bloomberg School of Public Health Baltimore Maryland USA; ^6^ Department of Neurology New York University Grossman School of Medicine New York New York USA; ^7^ Department of Neurology University of Mississippi Medical Center Jackson Mississippi USA; ^8^ Division of Epidemiology and Community Health School of Public Health, University of Minnesota Minneapolis Minnesota USA; ^9^ National Institute of Neurological Disorders and Stroke Intramural Research Program Bethesda Maryland USA; ^10^ Department of Biostatics, Epidemiology, and Informatics University of Pennsylvania Perelman School of Medicine Philadelphia Pennsylvania USA

**Keywords:** cohort study, epilepsy, stroke

## Abstract

**Objective:**

To estimate the risk of epilepsy associated with stroke in a community‐based cohort, with consideration of stroke type, number, and severity.

**Methods:**

Data from 15,100 Atherosclerosis Risk in Communities (ARIC) Study participants without stroke at baseline (1987–1989) were analyzed through 12/31/2022. Adjudicated stroke events were modeled as time‐varying exposures. Epilepsy was defined using International Classification of Diseases Ninth/Tenth Revisions codes. Adjusted Fine and Gray proportional hazards models were used to estimate the risk of epilepsy associated with stroke.

**Results:**

At baseline, the mean age of participants was 54 years, 55% were female, and 26% were of Black race. Over a median of 27 years, 1553 incident all‐cause strokes occurred. The risk of epilepsy was higher among individuals with versus without incident stroke (HR = 1.75, 95% CI = 1.50–2.04). There was evidence for interaction by age (*p*‐interaction = 0.03) whereby the risk of epilepsy associated with stroke was higher among individuals with younger versus older baseline age. Compared to no stroke, the point estimate for the risk of epilepsy associated with subarachnoid hemorrhage (HR = 2.94, 95% CI = 1.67–5.17) was higher than that for the risk of epilepsy associated with ischemic stroke (HR = 1.65, 95% CI = 1.40–1.94) and hemorrhagic stroke (HR = 1.47, 95% CI = 0.95, 2.27). The risk of epilepsy was similar by the number of incident strokes but was greater with increasing ischemic stroke severity.

**Interpretation:**

The risk of epilepsy was increased after an incident stroke. This work identifies high‐risk subgroups, including younger individuals, individuals with subarachnoid hemorrhage, and individuals with more severe ischemic strokes, who may benefit from closer clinical monitoring for seizures/epilepsy after a stroke.

## 1 Introduction

Stroke is one of the leading causes of death and disability in the United States (US) [1]. Seizures are an important complication post‐stroke and may occur immediately as a stroke symptom or manifest later as post‐stroke epilepsy (PSE; defined by the International League Against Epilepsy [ILAE] as one or more unprovoked seizures occurring ≥ 7 days post‐stroke [2]). It has been estimated that strokes account for approximately 11% of all adult‐onset epilepsy and up to 45% of epilepsy cases occurring in adults over 60 years of age [3]. The mechanisms of epilepsy after stroke differ depending on when the seizure occurs. Early‐onset seizures are often secondary to acute neuronal injury, glutamate excitotoxicity, and blood–brain barrier dysfunction, while late‐onset seizures (i.e., PSE) commonly result from gliotic scarring and chronic inflammation leading to neurodegeneration [4, 5, 6].

Several studies have examined risk factors associated with PSE. Hemorrhagic strokes [7], stroke severity [8], cortical strokes [9, 10, 11], and strokes occurring among younger individuals [7, 12] have all been suggested as factors related to higher PSE risk. Understanding risk for PSE is important to tailor counseling and identify high‐risk populations who may benefit from closer clinical monitoring for seizures/epilepsy after a stroke event. Limitations of much of the extant literature on PSE include limited follow‐up after stroke events and a lack of adjudicated stroke events.

In the present study, we aimed to estimate the risk of seizures/epilepsy associated with all stroke types in the deeply phenotyped Atherosclerosis Risk in Communities Study (ARIC) cohort with over 30 years of follow‐up. We hypothesized that individuals with incident all‐cause stroke would have a higher risk of subsequent seizures/epilepsy compared to individuals without stroke. In secondary analyses, we considered differences in the associations of all‐cause stroke with seizure/epilepsy risk by age, sex, and race, and considered the risk of seizure/epilepsy associated with stroke type (ischemic, hemorrhagic, subarachnoid hemorrhage), number of strokes, and ischemic stroke severity (defined by the National Institutes of Health Stroke Scale [NIHSS]). We hypothesized that individuals with incident hemorrhagic stroke and subarachnoid hemorrhage, with a greater number of stroke events, and with greater ischemic stroke severity would have a higher risk of subsequent seizures/epilepsy.

## 2 Methods

### 2.1 Study Population

The ARIC study is an ongoing community‐based prospective cohort study that included 15,792 adults aged 45 to 64 years at baseline (ARIC Visit 1, 1987–1989) who have been followed for over 30 years. Participants were recruited from 4 US communities: Forsyth County, North Carolina; northwest suburbs of Minneapolis, Minnesota; Washington County, Maryland; and Jackson, Mississippi [13]. Participants attended subsequent in‐person visits in 1990–1992 (Visit 2), 1993–1995 (Visit 3), 1996–1998 (Visit 4), 2011–2013 (Visit 5), 2016–2017 (Visit 6), 2018–2019 (Visit 7), 2020 (Visit 8), 2021–2022 (Visit 9), and 2023 (Visit 10), with continued follow‐up ongoing. Medical records from all hospitalizations occurring among ARIC participants in study communities are collected via continuous surveillance, and participants also report hospitalizations during annual (through 2011) and semi‐annual (starting in 2012) telephone follow‐up calls, and the corresponding records are obtained. Additionally, ARIC data are linked to Centers for Medicare & Medicaid Services (CMS) data (1991 to 2018) for participants aged 65 years and older enrolled in fee‐for‐service Part B.

**FIGURE 1:**
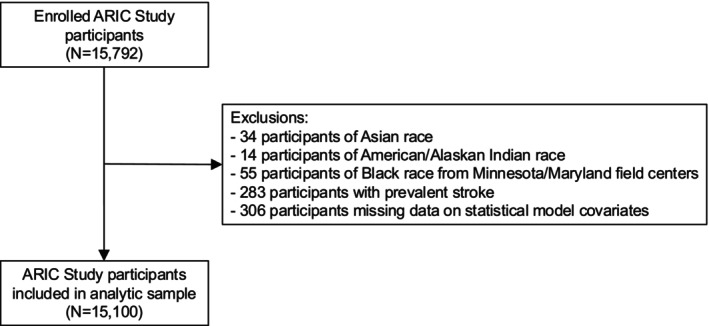
Flow chart of participant selection.

Of the 15,792 participants enrolled in the ARIC study in 1987–1989, we excluded 34 participants of self‐reported Asian race, 14 participants of self‐reported American/Alaskan Indian race, and 55 participants of self‐reported Black race from the Minnesota and Maryland centers due to small numbers and race/center aliasing, whereby mostly White participants were enrolled in Minnesota and Maryland and mostly Black and White participants were enrolled in North Carolina as a result of probability sampling of these communities, and only Black participants were enrolled in Mississippi [13]. We additionally excluded 283 participants with prevalent stroke at study baseline and 306 participants who were missing data on covariates included in statistical models, resulting in 15,100 included participants in our analytic sample (Figure 1).

The ARIC study was approved by institutional review boards at all participating institutions, and all participants (or legally authorized representatives) provided written informed consent at each study visit.

### 2.2 Stroke

Prior (prevalent) stroke was self‐reported at Visit 1 (these participants were excluded from the present analyses). Incident stroke events were modeled as a time‐varying exposure. Incident stroke events were identified using established methods through community surveillance of hospitalizations occurring in ARIC study communities and self‐report of hospitalizations during telephone follow‐up calls (after which medical records were obtained), and, for deaths, by linkage to State vital statistics registers and the National Death Index [14, 15, 16]. Possible stroke‐related hospital discharges were identified using International Classification of Diseases, Ninth Revision (ICD‐9) codes 430–438 and International Classification of Diseases, Tenth Revision (ICD‐10) codes G45.X, I60.X, I61.X, I62.X, I63.X, I65.X, I66.X, I67.X [17]. Stroke events were preliminarily classified by a computer‐generated algorithm [15], and were then reviewed by study physicians. Definite/probable all‐cause stroke events occurring through December 31, 2022 are the primary stroke exposure definition in this analysis. Classification as a definite/probable stroke required evidence of sudden or rapid onset of neurological symptoms lasting > 24 h or leading to death [13]. Potential incident strokes that were not hospitalized, including non‐hospitalized strokes with only death certificate diagnoses, were not included, as they could not be readily validated.

Secondary stroke exposure definitions include (1) stroke type as adjudicated by study physicians (ischemic [further stratified into ischemic stroke subtype in sensitivity analyses: thrombotic; lacunar; cardioembolic] [17]; hemorrhagic [intracerebral hemorrhage]; subarachnoid hemorrhage), (2) number of incident all‐cause strokes (0, 1, 2+), and (3) ischemic stroke severity ascertained by trained reviewers based on data from hospitalization records for all definite or probable ischemic strokes occurring through December 31, 2018, using a validated algorithm for retrospective National Institutes of Health Stroke Scale (NIHSS) scoring [18, 19]. Ischemic stroke severity was classified into 3 severity categories: low (NIHSS ≤ 5), medium (NIHSS 6–10), and high (NIHSS ≥ 11).

### 2.3 Seizure/Epilepsy and Post‐Stroke Seizure/Epilepsy (PSE)

Consistent with prior work [20, 21], incident seizure/epilepsy occurring after ARIC Visit 1 was derived from epilepsy‐ or seizure‐related ICD‐9/10 codes from continuously collected ARIC study hospitalization data and linked CMS data, including hospitalization, observation, emergency department, and outpatient encounters (ICD‐9 codes: 345.X, 780.39; ICD‐10 codes: G40.X, R56.9, Table S1). In our primary analyses, we defined seizure/epilepsy as at least one healthcare encounter with a seizure or epilepsy‐related ICD‐9/10 code and PSE was defined as at least 1 seizure or epilepsy‐related ICD‐9/10 code that occurred > 7 days after the stroke event [22]. Seizure/epilepsy‐related ICD‐9/10 codes occurring < 7 days from the stroke event were not considered PSE events; a lag of 7 days was implemented for post‐stroke person‐time in analyses. In sensitivity analyses, we defined seizure/epilepsy and PSE as requiring two or more healthcare encounters with a seizure or epilepsy‐related ICD‐9/10 code. In additional sensitivity analyses, we excluded individuals with a seizure/epilepsy diagnosis occurring within 2 years of study baseline (Visit 1) in order to exclude potential cases of prevalent seizure/epilepsy.

### 2.4 Covariates

Covariates included in statistical models were ascertained at ARIC Visit 1. Age (derived from birthdate), sex (male; female), race/center (Black Mississippi; Black North Carolina; White Maryland; White Minnesota; White North Carolina), education (< high school degree; high school degree, GED, or vocational school; some college or greater), smoking status (current; former; never), and alcohol consumption (current; former; never) were self‐reported. Diabetes was defined as fasting glucose ≥ 126 mg/dL, non‐fasting glucose ≥ 200 mg/dL, use of diabetes medications, or self‐report of a physician diagnosis of diabetes. Hypertension was defined as systolic blood pressure ≥ 140 mmHg, diastolic blood pressure ≥ 90 mmHg, or use of antihypertensive medications.

### 2.5 Statistical Analysis

**FIGURE 2:**
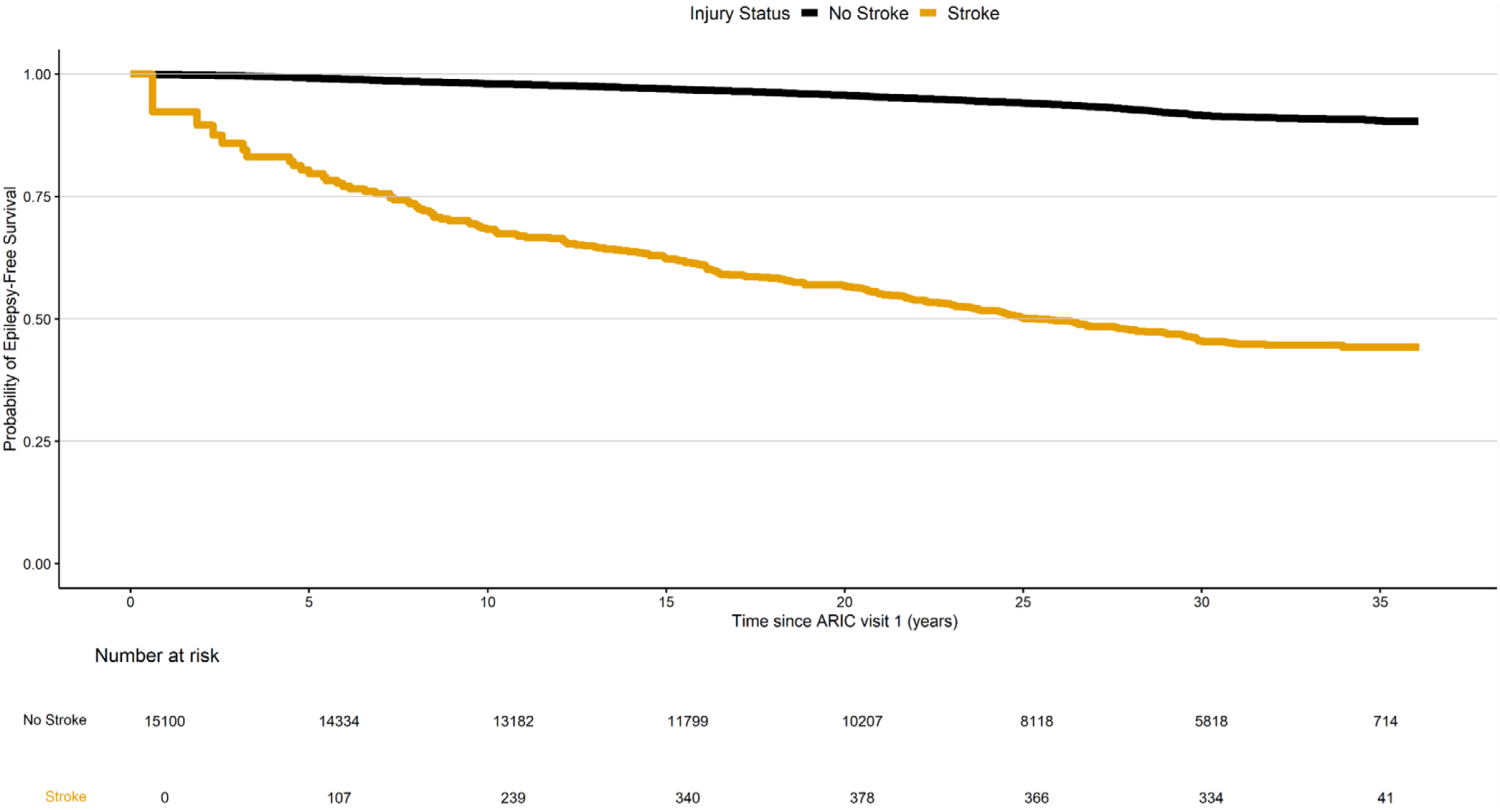
Cumulative seizure/epilepsy‐free survival by incident stroke status.

Participant characteristics are shown stratified by incident stroke status using means and standard deviations for continuous variables and numbers and percentages for categorical variables. Follow‐up extended from study enrollment (Visit 1; 1987–1989) until seizure/epilepsy, study withdrawal/loss to follow‐up, death, or administrative censoring on December 31, 2022 (for North Carolina, Minnesota, and Maryland sites) or December 31, 2020 (for Mississippi site). The administrative censoring date was defined based on the most updated ARIC hospital surveillance data available. Cumulative seizure/epilepsy free survival was estimated using Kaplan–Meier analyses. Fine and Gray proportional hazards models with time since enrollment as the time scale were used to estimate the associations between time‐varying stroke and risk of seizure/epilepsy, while accounting for the competing risk of mortality [23]. Kaplan–Meier (Figure 2) and complementary log–log plots (Figure S1) were used to confirm that the proportional hazards assumption was met [24]. All models were adjusted for age, sex, race/center, education, smoking status, alcohol consumption, diabetes, and hypertension. Formal testing for multiplicative interaction by age, sex, and race was performed using a Wald test.

Statistical analyses were performed using R, version 4.4.2 (R Foundation for Statistical Computing, Vienna, Austria) and statistical significance was defined a priori as a two‐sided *p* < 0.05.

## 3 Results

The mean baseline age of the 15,100 included participants (all initially free of stroke) was 54.1 years, 55.1% were female, and 26.1% self‐identified as Black race (Table 1). Throughout the median 27.1 (25th percentile, 75th percentile: 17.7, 33.0) year follow‐up period, 1523 (10.3%) participants had an incident stroke event (mean age at stroke: 73 years). Compared to individuals without stroke, individuals with incident stroke were slightly older at baseline (55.3 years vs. 54.0 years), more likely to be male (46.6% vs. 44.7%), have less than high school graduation (30.7% vs. 22.7%), have hypertension (47.8% vs. 32.9%) and have diabetes (19.8% vs. 10.9%).

**TABLE 1:** Participant characteristics, overall and stratified by incident stroke status, ARIC study Visit 1 (1987–1989).

	Overall (*N* = 15,100)	No incident stroke (*n* = 13,547)	Incident stroke^a^ (*n* = 1553)
Age (years), mean (SD)	54.1 (5.8)	54.0 (5.8)	55.3 (5.6)
Sex, *n* (%)			
Male	6778 (44.9)	6055 (44.7)	723 (46.6)
Female	8322 (55.1)	7492 (55.3)	830 (53.4)
Race/Center, *n* (%)			
Washington County, MD (White)	3857 (25.5)	3462 (25.6)	395 (25.4)
Minneapolis, MN (White)	3895 (25.8)	3554 (26.2)	341 (22.0)
Forsyth County, NC (White)	3412 (22.6)	3127 (23.1)	285 (18.4)
Forsyth County, NC (Black)	454 (3.0)	402 (3.0)	52 (3.3)
Jackson, MS (Black)	3482 (23.1)	3002 (22.2)	480 (30.9)
Education, *n* (%)			
Less than High School Degree	3550 (23.5)	3073 (22.7)	477 (30.7)
High School Degree	6182 (40.9)	5571 (41.1)	611 (39.3)
More than High School	5368 (35.5)	4903 (36.2)	465 (29.9)
Cigarette smoking, *n* (%)			
Current	3929 (26.0)	3479 (25.7)	450 (29.0)
Former	4882 (32.3)	4408 (32.5)	474 (30.5)
Never	6289 (41.6)	5660 (41.8)	629 (40.5)
Alcohol consumption, *n* (%)			
Current	8490 (56.2)	7697 (56.8)	793 (51.1)
Former	2855 (18.9)	2528 (18.7)	327 (21.1)
Never	3755 (24.9)	3322 (24.5)	433 (27.9)
Hypertension, *n* (%)	5205 (34.5)	4462 (32.9)	743 (47.8)
Diabetes, *n* (%)	1780 (11.8)	1472 (10.9)	308 (19.8)

^a^ Includes ischemic stroke, hemorrhagic stroke, and subarachnoid hemorrhage (24 individuals had both ischemic and hemorrhagic strokes; 4 had both ischemic stroke and subarachnoid hemorrhage).

A total of 1138 incident seizure/epilepsy diagnoses occurred over 369,253 person‐years of follow‐up. The median (25th percentile, 75 percentile) time between incident stroke and seizure/epilepsy diagnosis was 1.8 (0.7–5.8) years. The cumulative seizure/epilepsy‐free survival was consistently lower among individuals with incident stroke compared to those without (Figure 2). The unadjusted incidence rate of seizure/epilepsy was 2.86 per 1000 person‐years (95% CI 2.68, 3.05) among individuals without incident stroke and 20.32 per 1000 person‐years (95% CI 17.50, 23.46) among individuals with incident all‐cause stroke. After adjusting for covariates and the competing risk of death, incident all‐cause stroke was associated with 1.75 (95% CI 1.50–2.04) times the risk of seizure/epilepsy compared to no stroke (Table 2). This association was stronger among individuals who were younger compared to those older at study baseline (age less than median baseline age of 54 years, HR 1.97, 95% CI 1.54–2.53 versus age ≥ 54 years, HR 1.60, 95% CI 1.31–1.94, *p*‐interaction = 0.03). There was no evidence for interaction by sex or race (*p*‐interaction ≥ 0.65).

**TABLE 2:** Associations of incident stroke with risk of seizure/epilepsy.

	No. cases/PYs	IR (95% CI)	HR (95% CI)	*p*‐value for interaction
Overall				
No incident stroke	952/333,095	2.86 (2.68, 3.05)	1 (Reference)	
Incident stroke	186/9155	20.32 (17.50, 23.46)	1.75 (1.50, 2.04)	
Stratified by median baseline age				0.03
< 54 Years				
No incident stroke	379/182,179	2.08 (1.88, 2.30)	1 (Reference)	
Incident stroke	75/4019	18.66 (14.68, 23.39)	1.97 (1.54, 2.53)	
≥ 54 Years				
No incident stroke	573/150,915	3.80 (3.49, 4.12)	1 (Reference)	
Incident stroke	111/5136	21.61 (17.78, 26.02)	1.60 (1.31, 1.94)	
Stratified by sex				0.65
Female				
No incident stroke	518/192,911	2.69 (2.46, 2.93)	1 (Reference)	
Incident stroke	103/4560	22.59 (18.44, 27.39)	1.76 (1.43, 2.16)	
Male				
No incident stroke	434/140,184	3.1 (2.81, 3.40)	1 (Reference)	
Incident stroke	83/4595	18.07 (14.39, 22.39)	1.70 (1.35, 2.14)	
Stratified by Race				0.74
Black				
No incident stroke	328/77,354	4.24 (3.79, 4.72)	1 (Reference)	
Incident stroke	83/3254	25.51 (20.31, 31.62)	1.78 (1.41, 2.26)	
White				
No incident stroke	624/255,741	2.44 (2.25, 2.64)	1 (Reference)	
Incident stroke	103/5901	17.46 (14.25, 21.17)	1.69 (1.38, 2.07)	

*Note:* Fine–Gray proportional hazards model adjusted for stroke, age, sex, race‐center, education, diabetes, hypertension, smoking, alcohol consumption with death included as a competing risk.

In secondary analyses, the risk of seizure/epilepsy was examined in participants with versus without ischemic stroke, hemorrhagic stroke, and subarachnoid hemorrhage separately; characteristics are shown stratified by stroke type in Table S2. The point estimate for the risk of seizure/epilepsy associated with subarachnoid hemorrhage (HR 2.94, 95% CI 1.67–5.17) was higher than the point estimate for the risk of seizure/epilepsy associated with ischemic stroke (HR 1.65, 95% CI 1.40–1.94) and hemorrhagic stroke (HR 1.47, 95% CI 0.95, 2.27) (Table 3). Compared to no ischemic stroke, we observed a higher risk of seizure/epilepsy associated with greater ischemic stroke severity (NIHSS ≤ 5: HR 1.65, 95% CI 1.32–2.06, NIHSS 6–10: HR 2.69, 95% CI 2.00–3.62, and NIHSS ≥ 11: HR 3.31, 95% CI 2.46, 4.46). Results were similar across ischemic stroke subtypes (thrombotic; lacunar; cardioembolic) and by the number of all‐cause stroke events.

**TABLE 3:** Associations of incident stroke, number, type/subtype, and severity with risk of seizure/epilepsy.

	No. events/PYs	IR per 1000 PY (95% CI)	HR (95% CI)
Number of incident strokes			
0	1002/332,774	3.01 (2.83, 3.20)	1 (Reference)
1	143/6975	20.50 (17.28, 24.15)	1.27 (1.07, 1.51)
2+	22/1076	20.45 (12.82, 30.97)	1.17 (0.77, 1.76)
Incident hemorrhagic stroke			
No	1138/365,837	3.11 (2.93, 3.30)	1 (Reference)
Yes	19/473	40.14 (24.17, 62.68)	1.47 (0.95, 2.27)
Incident subarachnoid hemorrhage			
No	1152/368,063	26.91 (13.43, 48.15)	1 (Reference)
Yes	11/409	3.13 (2.95, 3.32)	2.94 (1.67, 5.17)
Incident ischemic stroke			
No	984/336,994	19.34 (16.47, 22.57)	1 (Reference)
Yes	161/8326	2.92 (2.74, 3.11)	1.65 (1.40, 1.94)
Incident ischemic stroke severity			
No incident ischemic stroke	946/322,579	2.93 (2.75, 3.13)	1 (Reference)
NIHSS ≤ 5	78/4921	15.85 (12.53, 19.78)	1.65 (1.32, 2.06)
NIHSS 6–10	41/1215	33.75 (24.22, 45.78)	2.69 (2.00, 3.62)
NIHSS ≥ 11	41/524	78.18 (56.10, 106.06)	3.31 (2.46, 4.46)
Incident ischemic stroke subtype			
Thrombotic			
No	1060/353,860	19.23 (15.32, 23.84)	1 (Reference)
Yes	83/4316	3.00 (2.82, 3.18)	1.63 (1.31, 2.02)
Lacunar			
No	1114/361,206	14.40 (9.65, 20.68)	1 (Reference)
Yes	29/2014	3.08 (2.91, 3.27)	1.25 (0.87, 1.78)
Cardioembolic			
No	1097/357,727	3.07 (2.89, 3.25)	1 (Reference)
Yes	46/1943	23.67 (17.33, 31.58)	1.31 (0.99, 1.75)

*Note:* Fine–Gray proportional hazards model adjusted for stroke, age, sex, race‐center, education, diabetes, hypertension, smoking, alcohol consumption with death included as a competing risk.

In sensitivity analyses restricting the definition of seizure/epilepsy to 2+ healthcare encounters with a seizure/epilepsy diagnostic code (instead of 1 healthcare encounter) (Table S3) and in sensitivity analyses excluding 227 individuals with prevalent epilepsy, defined as a diagnosis of seizure/epilepsy occurring within 2 years of baseline (Table S4), results were largely similar to the primary analyses, but estimates were less precise.

## 4 Discussion

In this large longitudinal community‐based cohort study of adults followed from midlife to late‐life, after covariate adjustment and accounting for the competing risk of death, individuals with hospitalized all‐cause stroke had an approximately 1.7‐fold increase in seizure/epilepsy risk. We also identified several high‐risk subgroups, including younger individuals, individuals with subarachnoid hemorrhage, and individuals with more severe strokes, who may benefit from closer clinical monitoring for seizures post‐stroke.

Our estimation of the epilepsy incidence rate in the stroke population, both when looking at all‐cause stroke and when stratified by stroke type, was lower than prior reports [25]. The difference in estimates from our study and these prior studies [25] is likely multifactorial and may be related to differences in analytic methods (e.g., we accounted for the competing risk of mortality), differences in stroke and/or epilepsy definitions, and different populations. Accounting for the competing risk of mortality is a significant strength of our study as the risk of mortality is approximately 50% in the first 5 years following a stroke [26, 27]. A recent study conducted on a nationwide Danish cohort did account for the competing risk of mortality and, similar to our findings, reported a lower risk of PSE compared to much of the prior literature [25]; however, the follow‐up time for incident epilepsy was restricted to 2 years [28]. Indeed, our study with a median of 27 years of follow‐up has among the longest durations of follow‐up; prior studies had follow‐up periods of less than 15 years [9, 10, 28, 29, 30, 31, 32, 33, 34, 35]. Furthermore, many prior studies were drawn from administrative data which rely solely on ICD codes to identify both stroke and epilepsy diagnoses [10, 28, 33, 34, 35]. While our study did use ICD codes to identify seizure/epilepsy diagnoses, our study offers the added rigor of having our stroke exposure diagnoses adjudicated by study physicians.

Several risk factors have been identified for PSE, including stroke subtypes, stroke severity, stroke location, cortical involvement, and age, among others [36, 37]. In contrast to some of these previous studies, which demonstrated a stronger association of hemorrhagic stroke with PSE compared to ischemic stroke with PSE [28, 29, 30, 33, 34], our study suggested that participants with ischemic and intraparenchymal hemorrhagic strokes had a similarly elevated risk of PSE. Further, we found that the point estimate for the rate of PSE associated with subarachnoid hemorrhage was higher than that for the rate of PSE associated with ischemic and hemorrhagic strokes. Given the potential impact of mortality on PSE risk, particularly given the higher risk of mortality within the first 30 days post‐stroke among individuals with hemorrhagic versus ischemic stroke [38], further studies are needed to clarify the rate difference between stroke subtypes which account for the competing risk of mortality.

Our findings suggest that ischemic strokes of greater severity are associated with greater risk of PSE compared to ischemic strokes of lesser severity, which corroborates findings of earlier studies reporting greater risk of epilepsy after more severe strokes [29, 39, 40]. Given that larger lesion volume and greater number of stroke events have been reported as robust predictors of PSE in previous studies [41, 42, 43], we additionally hypothesized that recurrent stroke events would be associated with an increased risk of PSE. However, accounting for the competing risk of death, we did not observe any difference in epilepsy risk between participants who had one stroke event and those who had two or more stroke events. Within the limitations of a lower sample size for repeated strokes, our study suggested that the recurrence of stroke is not associated with higher risk for PSE.

Consistent with other studies [28, 30, 44], we found that younger age was associated with a greater risk of seizure/epilepsy after stroke compared to older age. It is important to note that we used the age at the time of enrollment rather than the age of stroke onset for our analyses as we included individuals without incident stroke events in our analytic sample, so the age groups for analyses may not be reflective of age of stroke and seizure/epilepsy onset. In addition, our study demonstrated that the risk of epilepsy associated with stroke was similar by sex. A recent Danish study reported a significantly higher PSE risk in males compared to females after ischemic stroke while no sex differences were observed after hemorrhagic strokes [28]. Few studies have investigated sex differences in the risk of PSE. Future studies should investigate potential sex differences in associations of stroke types with epilepsy risk; small numbers of participants with hemorrhagic stroke and subarachnoid hemorrhage precluded further stratification by sex in our analyses. We found no significant differences in associations of stroke with seizure/epilepsy risk between self‐reported Black and White race groups. Limited studies including race stratified results have suggested that individuals of non‐White race have a higher PSE risk compared to individuals of White race [34].

Our results should be interpreted in the context of several limitations. First, while all hospitalized stroke events were adjudicated in the ARIC study by physicians, ICD codes alone were used to identify the diagnoses of seizure/epilepsy, which could lead to misclassification of our outcomes, particularly for non‐motor seizures. However, our definition of seizure/epilepsy and PSE is consistent with prior literature [45], and we conducted a sensitivity analysis defining seizure/epilepsy as ICD codes for seizure/epilepsy from at least two medical encounters, which had similar results to our primary seizure/epilepsy definition that required an ICD code for seizure/epilepsy at one or more medical encounters. Epilepsy defined as two or more medical encounters with a seizure/epilepsy‐related ICD code has been shown to have a sensitivity of 94% and specificity of 91% [46]. We additionally performed sensitivity analyses excluding individuals with seizure/epilepsy occurring within the first 2 years of follow‐up to avoid including prevalent seizure/epilepsy, as the ARIC study does have self‐report of seizures/epilepsy at baseline; these results were also similar to our primary analysis. Second, analyses of associations of hemorrhagic stroke and subarachnoid hemorrhage with incident epilepsy were limited by small sample size. Additionally, we did not have detailed information on certain stroke characteristics, so we were unable to consider factors such as affected vascular territory and cortical versus non‐cortical location, among others, limiting our ability to compare our results to existing PSE calculators such as the SeLECT score [40]. Ischemic stroke subtypes in the ARIC study are classified as thrombotic, lacunar, cardioembolic [17]. This classification categorizes embolic strokes of undetermined source as thrombotic strokes, and we are unable to distinguish between these groups as could be done using other classification systems such as the Trial of Org 10,172 in Acute Stroke Treatment (TOAST) system [47]. Stroke severity, as assessed by the NIHSS, was available only for ischemic strokes, so we were not able to evaluate associations of hemorrhagic stroke or subarachnoid hemorrhage severity with risk of incident epilepsy. Similarly, we lacked detailed information on seizure semiology, frequency, and severity. Finally, we did not have information on antiseizure medication use in the time following the stroke events, as early prophylactic use of antiseizure medication in the stroke population might lower the incidence of PSE. However, prophylactic antiseizure medication in post‐stroke patients is not recommended by guidelines [48, 49].

## 5 Conclusions

In conclusion, in this community‐based population, the risk of epilepsy was 1.7 times higher among middle‐aged to older adults with an incident all‐cause stroke versus without, after accounting for confounders and the competing risk of death. Our study identified several high‐risk subgroups who may benefit from closer clinical monitoring for seizures/epilepsy after a stroke event, including younger individuals, individuals with subarachnoid hemorrhage, and individuals with more severe strokes.

## Author Contributions

Conception and design of the study: J.Z., A.A.L., C.A.L., E.J., and A.L.C.S. Acquisition, analysis, and interpretation of data: J.Z., A.A.L., C.A.L., E.J., A.L.C.S., M.C.J., A.R., S.K., S.K., J.H., K.L., and R.F.G. Drafting a significant portion of the manuscript: J.Z., A.A.L., C.A.L., and A.L.C.S. Critical revision of the manuscript: J.Z., A.A.L., C.A.L., E.J., A.L.C.S., M.C.J., A.R., S.K., S.K., J.H., K.L., R.F.G.

## Conflicts of Interest

The authors declare no conflicts of interest.

## Supporting information


Data S1.

## Data Availability

ARIC study data are available via the Biologic Specimen and Data Repository Information Coordinating Center (BioLINCC) (https://biolincc.nhlbi.nih.gov/home/).
