# Supplementary material for: Associations of Stroke With Risk of Epilepsy: Results From the Atherosclerosis Risk in Communities (ARIC) Study
Source: Ann Clin Transl Neurol. 2025 Jul 15;12(10):2107–15. doi: 10.1002/acn3.70144 (PMC12516231; doi:10.1002/acn3.70144)

Supplemental Material

Zhou J, Ladak AA, Law CA, Johansen MC, Reyes A, Koton S, Kelly S, Huang J, Lakshminarayan K, Gottesman RF, Johnson E, Schneider ALC. Associations of Stroke with Risk of Epilepsy: Results from the Atherosclerosis Risk in Communities (ARIC) Study.

Table of Contents

eTable 1. ICD-9/10 Codes Used to Define Seizure/Epilepsy 2

eTable 2. Participant Characteristics Stratified by Incident Stroke Type, ARIC Study Visit 1 (1987-1989) 3

eTable 3. Associations of Incident Stroke with Risk of Seizure/Epilepsy (Requiring 2 or More Medical Encounters with a Seizure/Epilepsy-Related ICD Code to Define Seizure/Epilepsy) 4

eTable 4. Associations of Incident Stroke with Risk of Seizure/Epilepsy Excluding 227 Individuals with Seizure/Epilepsy Occurring in the First 2-Years of Follow-up 5

eFigure 1. Complementary Log-Log Plot for Proportional Hazards Assumption 6

eTable 1. ICD-9/10 Codes Used to Define Seizure/Epilepsy.

ICD-9 Codes	ICD-10 Codes
345.0x Generalized nonconvulsive epilepsy<br>345.1x Generalized convulsive epilepsy<br>345.2 Petit mal status<br>345.3 Grand mal status<br>345.4x Localization-related (focal) (partial) epilepsy with complex partial seizures<br>345.5x Localization-related (focal) (partial) epilepsy with simple partial seizures<br>345.7x Epilepsia partialis continua<br>345.8x Other forms of epilepsy and recurrent seizures<br>345.9x Epilepsy unspecified<br>780.39 Other convulsions	G40.0xx Localization-related (focal) (partial) epilepsy<br>G40.1xx Localization-related (focal) (partial) epilepsy with complex partial seizures<br>G40.2xx Localization-related (focal) (partial) epilepsy with simple partial seizures<br>G40.3xx Generalized idiopathic epilepsy<br>G40.4xx Other generalized epilepsy and epileptic syndromes<br>G40.8xx Other epilepsy and recurrent seizures<br>G40.9xx Epilepsy, unspecified<br>R56.1 - Post-traumatic seizures<br>R56.9 Seizure (convulsive), convulsions NOS

References:

1. Johnson EL, Krauss GL, Kucharska-Newton A, et al. Dementia in late-onset epilepsy: The Atherosclerosis Risk in Communities study. *Neurology*. Dec 15 2020;95(24):e3248-e3256.
2. Schneider ALC, Gottesman RF, Krauss GL, et al. Association of Head Injury With Late-Onset Epilepsy: Results From the Atherosclerosis Risk in Communities Cohort. *Neurology*. Feb 22 2022;98(8):e808-e817.

eTable 2. Participant Characteristics Stratified by Incident Stroke Type, ARIC Study Visit 1 (1987-1989).

	No Incident Stroke (n=13,547)	Incident Ischemic Stroke (n=1,364)	Incident Hemorrhagic Stroke (n=156)	Incident Subarachnoid Hemorrhage (n=51)
Age (years), mean (SD)	54.0 (5.8)	55.3 (5.6)	54.5 (5.7)	54.4 (5.4)
Sex, n (%)				
Male	6,055 (44.7)	654 (48.0)	67 (42.9)	13 (25.5)
Female	7,492 (55.3)	710 (52.0)	89 (57.1)	38 (74.5)
Race/Center, n (%)				
Washington County, MD (White)	3,462 (25.6)	341 (25.0)	45 (28.8)	12 (23.5)
Minneapolis, MN (White)	3,554 (26.2)	308 (22.6)	27 (17.3)	9 (17.6)
Forsyth County, NC (White)	3,127 (23.1)	247 (18.1)	27 (17.3)	12 (23.5)
Forsyth County, NC (Black)	402 (3.0)	49 (3.6)	4 (2.6)	0 (0)
Jackson, MS (Black)	3,002 (22.2)	419 (30.7)	53 (34.0)	18 (35.3)
Education, n (%)				
Less than High School Degree	3,073 (22.7)	431 (31.6)	41 (26.3)	14 (27.5)
High School Degree	5,571 (41.1)	522 (38.3)	70 (44.9)	24 (47.1)
More than High School	4,903 (36.2)	411 (30.1)	45 (28.8)	13 (25.5)
Cigarette Smoking, n (%)				
Current	3,479 (25.7)	395 (29.0)	42 (26.9)	16 (31.4)
Former	4,408 (32.5)	427 (31.3)	44 (28.2)	12 (23.5)
Never	5,660 (41.8)	542 (39.7)	70 (44.9)	23 (45.1)
Alcohol Consumption, n (%)				
Current	7,697 (56.8)	698 (51.2)	77 (49.4)	27 (52.9)
Former	2,528 (18.7)	291 (21.3)	35 (22.4)	7 (13.7)
Never	3,322 (24.5)	375 (27.5)	44 (28.2)	17 (33.3)
Hypertension, n (%)	4,462 (32.9)	661 (48.5)	75 (48.1)	20 (39.2)
Diabetes, n (%)	1,472 (10.9)	288 (21.1)	20 (12.8)	3 (5.9)

Note: 18 individuals had both ischemic *and* hemorrhagic strokes; 20 individuals had both ischemic stroke *and* subarachnoid hemorrhage.

eTable 3. Associations of Incident Stroke with Risk of Seizure/Epilepsy (Requiring 2 or More Medical Encounters with a Seizure/Epilepsy-Related ICD Code to Define Seizure/Epilepsy).

	No. Events / PYs	IR per 1,000 PY (95% CI)	HR (95% CI)	P-value for Interaction
Overall				
No Incident Stroke	660 / 336,628	1.98 (1.83, 2.14)	1 (Reference)	
Incident Stroke	134 / 9,392	14.27 (11.95, 16.90)	1.78 (1.48, 2.14)	
Stratified by Median Baseline Age				0.24
<54 Years				
No Incident Stroke	264 / 182,098	1.45 (1.28, 1.64)	1 (Reference)	
Incident Stroke	53 / 4,122	12.86 (9.63, 16.82)	1.94 (1.44, 2.61)	
≥54 Years				
No Incident Stroke	396 / 151,531	2.61 (2.36, 2.88)	1 (Reference)	
Incident Stroke	81 / 5,269	15.37 (12.21, 19.11)	1.65 (1.31, 2.09)	
Stratified by Sex				0.73
Female				
No Incident Stroke	366 / 193,115	1.90 (1.71, 2.10)	1 (Reference)	
Incident Stroke	71 / 4,681	15.17 (11.85, 19.13)	1.67 (1.30, 2.15)	
Male				
No Incident Stroke	294 / 140,514	2.09 (1.86, 2.35)	1 (Reference)	
Incident Stroke	63 / 4,711	13.37 (10.28, 17.11)	1.89 (1.44, 2.47)	
Stratified by Race				0.62
Black				
No Incident Stroke	236 / 77,659	3.04 (2.66, 3.45)	1 (Reference)	
Incident Stroke	63 / 3,336	18.88 (14.51, 24.16)	1.86 (1.41, 2.45)	
White				
No Incident Stroke	424 / 255,970	1.66 (1.50, 1.82)	1 (Reference)	
Incident Stroke	71 / 6,055	11.73 (9.16, 14.79)	1.69 (1.32, 2.16)	

Fine-Gray proportional hazards model adjusted for stroke, age, sex, race-center, education, diabetes, hypertension, smoking, alcohol consumption with death included as a competing risk.

eTable 4. Associations of Incident Stroke with Risk of Seizure/Epilepsy Excluding 227 Individuals with Seizure/Epilepsy Occurring in the First 2-Years of Follow-up.

	No. Events / PYs	IR per 1,000 PY (95% CI)	HR (95% CI)	P-value for Interaction
Overall				
No Incident Stroke	947 / 332,920	20.61 (17.70, 23.87)	1 (Reference)	
Incident Stroke	179 / 8,683	2.84 (2.67, 3.03)	1.70 (1.46, 1.99)	
Stratified by Median Baseline Age				0.03
<54 Years				
No Incident Stroke	378 / 182,137	2.08 (1.87, 2.30)	1 (Reference)	
Incident Stroke	73/3,810	19.16 (15.02, 24.09)	1.95 (1.52, 2.50)	
≥54 Years				
No Incident Stroke	569 / 150,784	3.77 (3.47, 4.10)	1 (Reference)	
Incident Stroke	106 / 4,874	21.75 (17.81, 26.31)	1.55 (1.27, 1.90)	
Stratified by Sex				0.82
Female				
No Incident Stroke	516 / 192,843	2.68 (2.45, 2.92)	1 (Reference)	
Incident Stroke	98 / 4,372	22.42 (18.20, 27.32)	1.70 (1.37, 2.10)	
Male				
No Incident Stroke	431 / 140,078	3.08 (2.79, 3.38)	1 (Reference)	
Incident Stroke	81 / 4,312	18.79 (14.92, 23.35)	1.69 (1.34, 2.13)	
Stratified by Race				0.94
Black				
No Incident Stroke	326 / 77,281	4.22 (3.77, 4.70)	1 (Reference)	
Incident Stroke	78 / 3,035	25.70 (20.32, 32.08)	1.70 (1.34, 2.16)	
White				
No Incident Stroke	621 / 255,639	2.43 (2.24, 2.63)	1 (Reference)	
Incident Stroke	101 / 5,649	17.88 (14.56, 21.73)	1.68 (1.37, 2.06)	

Fine-Gray proportional hazards model adjusted for stroke, age, sex, race-center, education, diabetes, hypertension, smoking, alcohol consumption with death included as a competing risk.

eFigure 1. Complementary Log-Log Plot for Proportional Hazards Assumption.

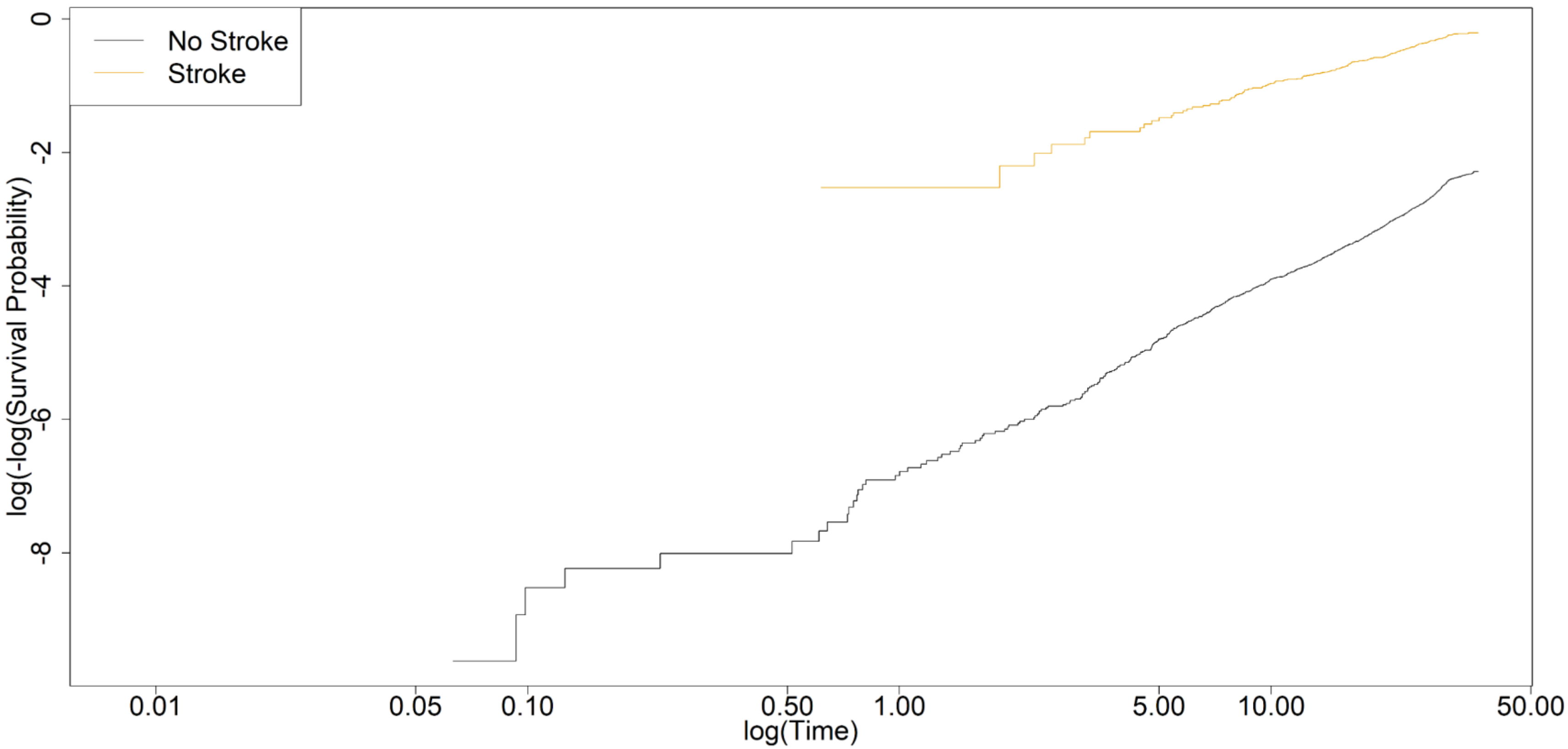

Supplement: Supplementary file 1 — Data S1. [file ACN3-12-2107-s001.pdf]
